# Supplementary material for: Extracellular modulation of TREK-2 activity with nanobodies provides insight into the mechanisms of K2P channel regulation
Source: Nat Commun. 2024 May 16;15:4173. doi: 10.1038/s41467-024-48536-2 (PMC11099193; doi:10.1038/s41467-024-48536-2)
Supplement: Supplementary file 1 — Supplementary Information [file 41467_2024_48536_MOESM1_ESM.pdf]

Table S1. X-ray data collection and refinement statistics.

	TREK-2 Nb58 (8QZ1)	TREK-2 Nb61 (8QZ2)	TREK-2 Nb67 (8QZ3)	TREK-2 Nb76 (8QZ4)
**Data collection**				
Space group	H3	$C222_1$	$P22_12_1$	$P2_12_12_1$
Cell dimensions				
a, b, c (Å)	233.663, 233.663, 67.338	56.538, 111.698, 289.208	51.264, 102.100, 215.817	68.280, 75.756, 259.150
α, β, γ (°)	90, 90, 120	90, 90, 90	90, 90, 90	90, 90, 90
Resolution (Å)	44.16-3.59 (3.69-3.59)	57.84-3.50 (3.58-3.50)	58.81-2.40 (2.49-2.40)	51.83-3.20 (3.34-3.20)
No. reflections / unique	73,209 / 14,181	63,807 / 11,445	221,034 / 34,388	72,190 / 17,904
R_{pim}	0.051 (0.649)	0.223 (0.799)	0.087 (0.536)	0.066 (0.522)
R_{meas}	0.116 (1.339)	0.540 (1.997)	0.219 (1.363)	0.134 (1.065)
I/σI	8.6 (1.3)	3.7 (1.2)	6.3 (1.6)	8.4 (1.7)
Correlation coefficient ($CC_{1/2}$)	0.996 (0.511)	0.997 (0.488)	0.989 (0.610)	0.998 (0.615)
Completeness (%)	88.6 (55.7)	95.0 (79.7)	75.6 (35.9)	77.9 (34.4)
Multiplicity	5.2 (4.1)	5.6 (6.1)	6.4 (6.3)	4.0 (4.0)
**Refinement**				
Resolution (Å)	31.30-3.60	52.98-3.50	58.81-2.40	31.25-3.20
No. reflections	14,165	11,398	34,319	17,829
R_{work} / R_{free} (%)	25.51 / 29.84	27.95 / 30.82	27.75 / 29.09	27.04 / 29.84
No. atoms				
Protein	5132	4507	6878	5321
Ligand/ion	3	4	13	144
Water	-	-	191	8
B-factors				
Protein	154.18	65.65	44.79	76.08
Ligand/ion	138.86	41.13	47.07	144.17
Water	-	-	28.52	26.30
R.m.s. deviations				
Bond lengths (Å)	0.0053	0.0049	0.0046	0.0044
Bond angles (°)	0.708	0.684	0.679	0.655

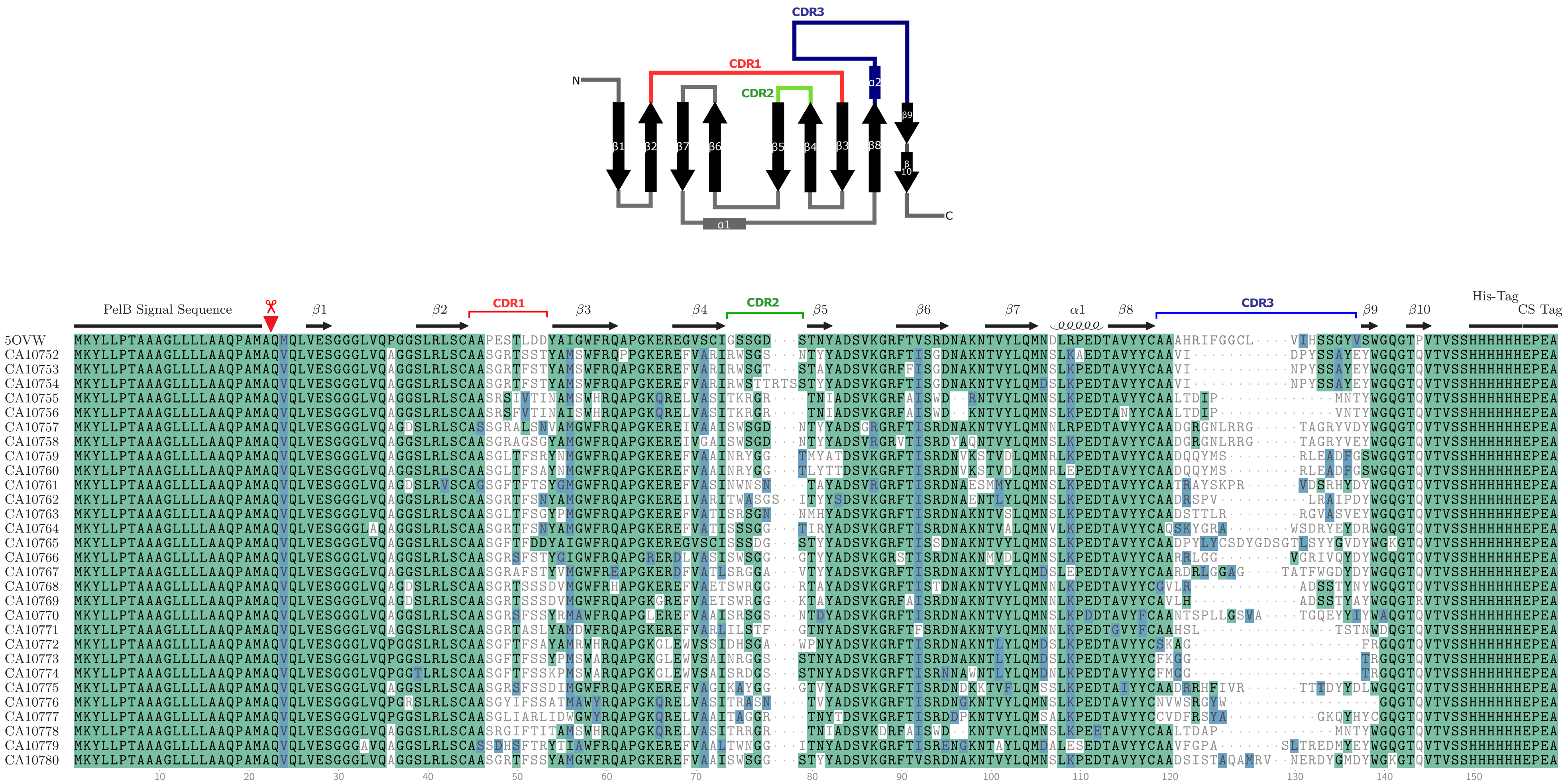

Figure S1 (Top) Cartoon description of a typical nanobody structure indicating the β-sheet structure and CDR loops. Below is a sequence alignment with the TREK-2 nanobodies used in this study. The β-sheets, CDR1, CDR2 and CDR3 loops are highlighted. The PelB signal sequence for periplasmic secretion and His-Tag for purification are also included in these sequences. The purified nanobodies are cleaved at the position indicated (red inverted triangle and scissors). The numbering of residues within each nanobody therefore starts from this point.

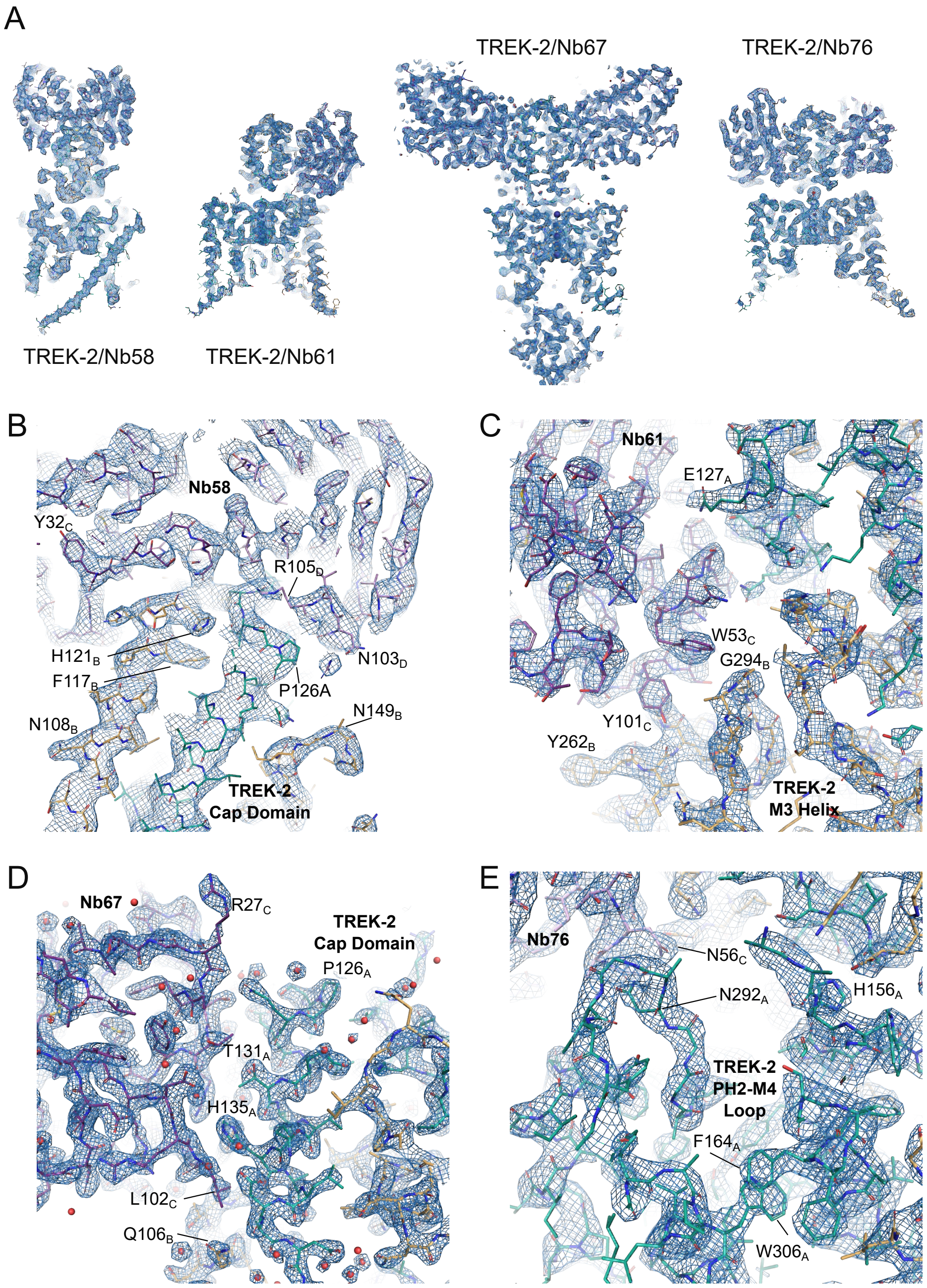

Figure S2. Quality of electron density for different TREK-2/nanobody complexes. **A**) Slice though of density for the four structures indicated. **B**) Interactions of Nb-Binder-58 with the Cap domain of TREK-2. **C**) Interactions of Nb-Inhibitor-61 with the top of M3 and the Cap domain. **D**) Interactions of Nb-Activator-67 with the Cap domain. **E**) Interactions of Nb-Activator-76 with the P2-M4 loop and Cap domain.

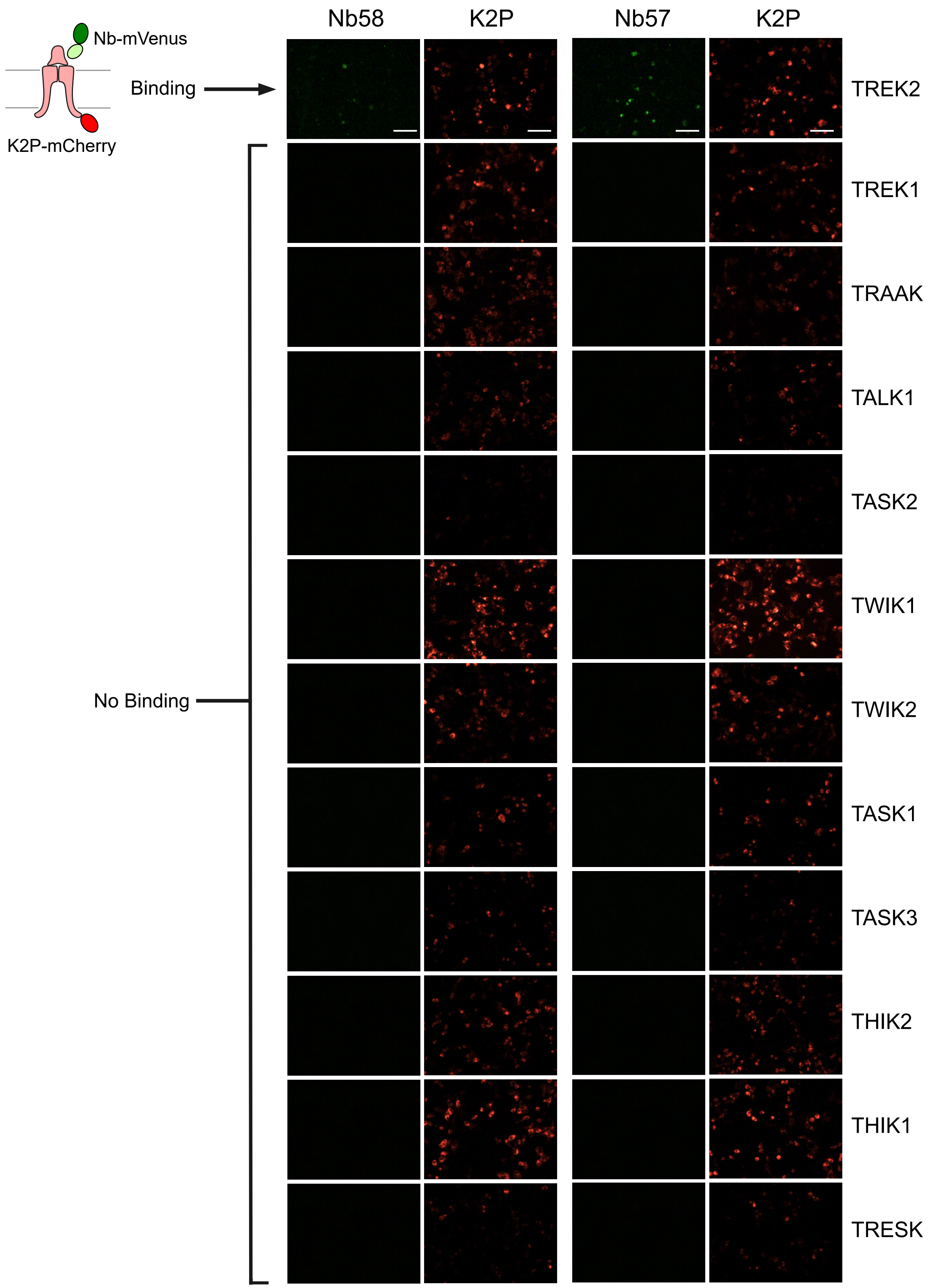

Figure S3: Nanobody binding to TREK-2 expressed in HEK293 cells. This data complements the data shown in Figure 2B and demonstrates the specificity of Nb57 and Nb58 binding to TREK-2 channels and no other K2P channels examined. The cartoon in the top left summarises the binding assay: the K2P channels are fused to mCherry and the nanobodies to mVenus. Binding is only detected with TREK2 and not with any of the other K2P channels tested. The overlays for Nb57 and Nb58 are shown in greater detail in Figure 2B.

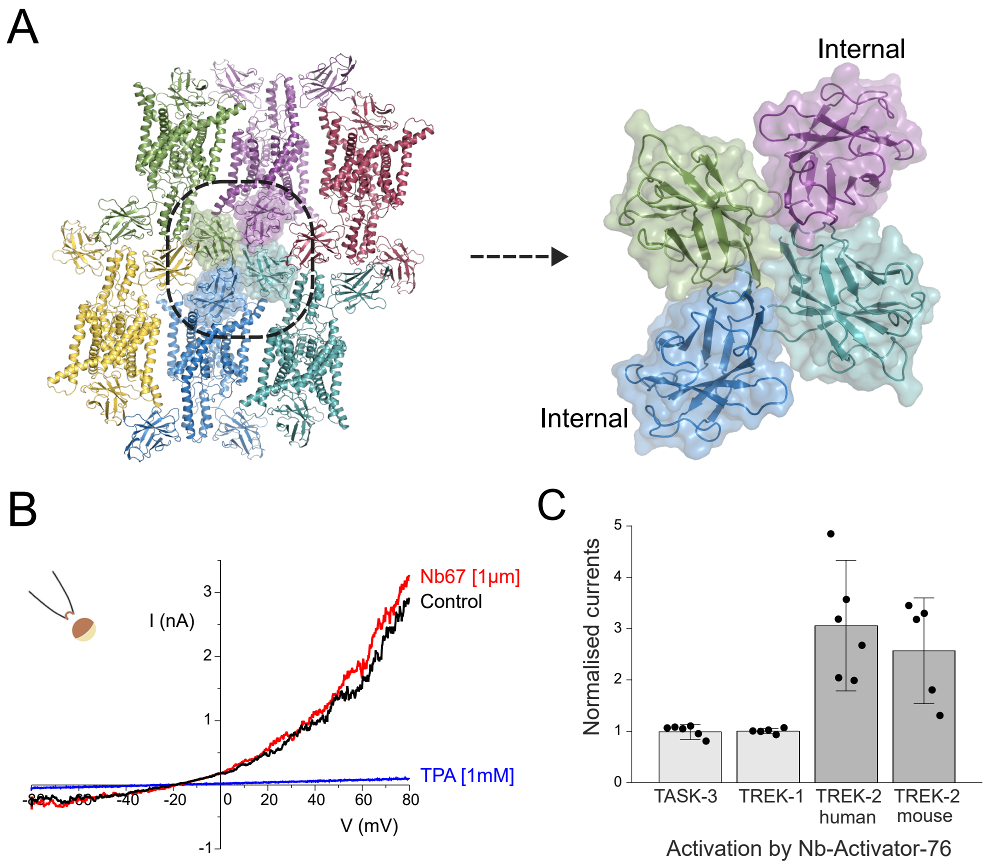

Figure S4. A) Crystal packing for the Nb67/TREK-2 complex showing that the 'internally' bound copy of Nb67 (blue/purple) forms tetramers with the extrenally bound copies within the unit cell. The area within this dotted line is expanded on the right-hand side showing this tetramerisation more clearly. **B**) Intracellular application of Nb67 has no functional effect. Representative traces showing Nb67 perfused onto the intracellular surface of giant patches excised from oocytes expressing TREK-2 channels. The same channels could be inhibited by intracellular application of tetrapentylammonium (TPA). **C**) Normalised whole-cell currents showing that Nb76 (1 μM) does not activate TASK-3 or TREK-1. However, marked activation of both human and mouse TREK-2 currents are observed (Error bars represent mean ± S.D).

A

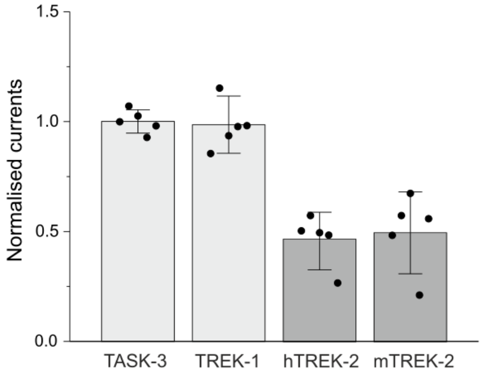

B

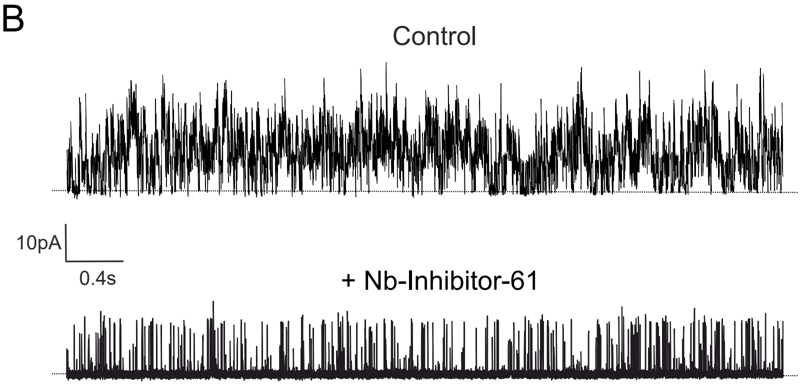

Figure S5. A. Specificity of Nb61. Normalised whole-cell currents showing that Nb61 does not inhibit either TASK-3 or TREK-1 but that it does inhibit both mouse and human TREK-2 (Error bars represent mean ± S.D) **B.** The effect of Nb-Inhibitor-61 on TREK-2 activity at the single channel level. Due to the low intrinsic open-probability of TREK-2, a qualitative comparison is shown above between the behaviour of multi-channel patches with (bottom) or without (top) Nb61 included in the pipette solution. Suppression of TREK-2 activity is seen by a reduction in the durations of bursts of openings, and increase in the time spent in the long closed states. This is consistent with the idea that this nanobody acts directly as a slow blocker resulting in a complete block of K^+ flux through the channel.

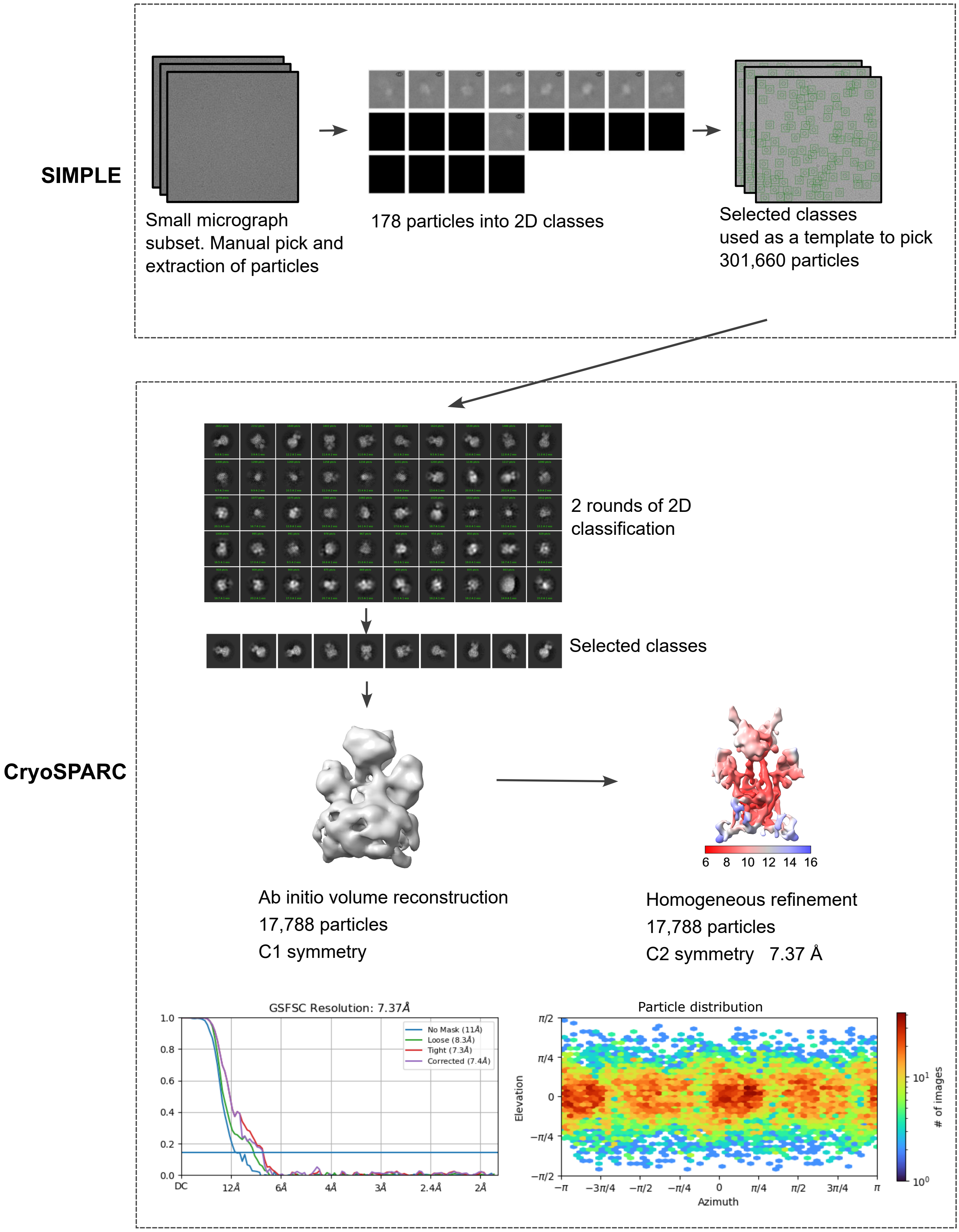

Figure S6. Flowchart for the single particle reconstruction of TREK-2 in complex with the biparatopic linked Nb-inhibitor-5861. Particles were picked and extracted in SIMPLE. 2D classification, volume reconstruction and refinement were carried out in CryoSPARC. The final reconstruction of 17,788 particles gave a map of resolution 7.37 Å.
